# Trust calibration and perceived developmental gains in university students' collaboration with generative AI: an exploratory sequential mixed methods study

**DOI:** 10.3389/fpsyg.2026.1861072

**Published:** 2026-09-25

**Authors:** Fan Bu, Wei Li

**Affiliations:** 1 School of Marxism, Heilongjiang University, Harbin, Heilongjiang, China; 2 Yangzhou University, Yangzhou, China

**Keywords:** AI literacy, collaborative regulation, generative artificial intelligence, higher education, mixed methods research, self-regulated learning, trust calibration

## Abstract

**Introduction:**

Research on generative artificial intelligence (GenAI) in higher education has expanded rapidly, but the field still explains student adoption more clearly than it explains how AI supported activity becomes higher quality learning. Prior studies have tended to emphasize use intention, broad AI literacy, ethical concern, or general perceptions of impact, while offering less precise accounts of the process linking supportive educational contexts to students' trust judgments, regulatory behavior, and developmental outcomes.

**Methods:**

To address this gap, the present study proposes and tests a contextual support, trust calibration, collaborative regulation, and perceived transfer gains model using an exploratory sequential mixed methods design. In Study 1, semi structured interviews with university students were analyzed through open, axial, and selective coding to identify the core dimensions of the model and to inform item development. In Study 2, a 22 item instrument was pilot tested and then administered to 642 students for confirmatory factor analysis and structural equation modeling.

**Results and discussion:**

The structural model showed that contextual support positively predicted trust calibration and collaborative regulation; trust calibration positively predicted collaborative regulation and perceived transfer gains; and collaborative regulation showed the strongest association with perceived transfer gains. The direct path from contextual support to perceived transfer gains was not statistically significant, whereas the chained indirect pathway through trust calibration and collaborative regulation was significant. The findings suggest that developmental benefits in students' collaboration with GenAI are better understood as theory consistent structural relationships shaped by contextual guidance, calibrated trust, and active regulation rather than as automatic consequences of frequent AI use. The study contributes a more differentiated process model for GenAI supported learning in higher education and clarifies the interpretive limits of subjective cross sectional outcome measures.

## 1 Introduction

Generative artificial intelligence (GenAI) has rapidly become embedded in university students' everyday academic work. Students use conversational systems to search for information, generate and organize ideas, plan essays, revise drafts, summarize readings, produce code, and explore alternative explanations (O'Dea, 2024; Nguyen et al., 2024; Atchley et al., 2024). As GenAI becomes increasingly involved in task interpretation, idea development, evidence selection, and revision, it is no longer merely a background information tool; rather, it can function as an interactive partner that influences how students approach academic tasks and distribute responsibility between their own reasoning and AI-generated contributions (Jeon and Lee, 2023; Nguyen et al., 2024; Darvishi et al., 2024). However, the educational significance of this development cannot be inferred from access, frequency of use, or faster task completion alone. The more important educational issue is whether collaboration with GenAI supports students' agency, higher-order thinking, self-regulated learning, and ability to transfer effective strategies across tasks, or instead encourages superficial engagement and the outsourcing of cognitive effort (Zimmerman, 2000; Darvishi et al., 2024; Lee et al., 2024; Weng et al., 2024; Zhou et al., 2024).

This is also a psychological issue because the quality of GenAI-supported learning depends on how students judge the reliability and limitations of AI output, monitor uncertainty, allocate cognitive responsibility, and regulate their behavior during interaction. Excessive trust may lead students to accept fluent but inaccurate responses without sufficient scrutiny, whereas indiscriminate distrust may prevent them from benefiting from useful scaffolding (Lee and See, 2004; Nazaretsky et al., 2025; Lelescu et al., 2025). Moreover, students differ substantially in whether they verify claims, compare AI output with external evidence, refine prompts, and revise generated content before incorporating it into their work (Nguyen et al., 2024; Mart́ın-Moncunill and Alonso Mart́ınez, 2025). These differences suggest that the developmental value of GenAI does not arise automatically from use itself, but depends on the psychological judgments and regulatory processes through which students manage human–AI collaboration. This leads to the central psychological and educational question motivating the present study: under what conditions does collaboration with GenAI strengthen students' judgment, reflection, and strategy transfer rather than merely accelerate task completion?

Several strands of prior research provide partial answers to this question. One stream focuses on adoption and institutional integration, showing that the use of GenAI in higher education is influenced by perceived usefulness, social norms, policy clarity, and disciplinary expectations (Almogren et al., 2024; Venkatesh et al., 2003), (O'Dea, 2024; Kurtz et al., 2024). A second stream examines AI literacy and ethical preparedness, emphasizing that students need not only operational familiarity but also evaluative and ethical competence when using AI in academic settings (Ng et al., 2021; Chen et al., 2025), (Saleh and ElSayary, 2025). A third stream studies human AI collaboration in learning and writing, demonstrating that students' interactions with AI vary substantially in depth, purpose, and degree of critical engagement (Jeon and Lee, 2023; Nguyen et al., 2024), (Atchley et al., 2024). A fourth stream highlights the roles of trust, verification, and self-regulated learning in explaining whether students rely on AI productively or uncritically (Lee and See, 2004; Weng et al., 2024; Lee et al., 2024; Nazaretsky et al., 2025; Mart́ın-Moncunill and Alonso Mart́ınez, 2025).

Despite this progress, the literature remains conceptually fragmented in at least three ways. Adoption oriented studies often explain why students use GenAI, but not how usage is transformed into higher quality learning activity (O'Dea, 2024; Kurtz et al., 2024). AI literacy research often identifies important competence dimensions, but it does not always specify the proximal process through which literacy relevant judgments become regulatory behavior in actual task performance (Ng et al., 2021; Chen et al., 2025), (Saleh and ElSayary, 2025). Trust research in educational AI is expanding, yet the boundary between general trust, warranted reliance, critical evaluation, and task specific verification is still theoretically unsettled (Lelescu et al., 2025; Nazaretsky et al., 2025). Related work on student interaction patterns also shows that learners vary markedly in how they verify, compare, and revise AI output during academic tasks (Nguyen et al., 2024; Atchley et al., 2024). These gaps matter because frequent GenAI use does not reliably produce the same developmental outcomes across students: some students question, verify, compare, and revise AI output, whereas others accept fluent responses too readily and outsource cognitive effort.

The present study addresses this gap by proposing a process model linking contextual support, trust calibration, collaborative regulation, and perceived transfer gains. Contextual support refers to the external instructional environment surrounding GenAI use, including teacher guidance, assessment design, peer modeling, and institutional norms (O'Dea, 2024; Kurtz et al., 2024). Trust calibration refers to the degree to which students' trust in AI is appropriately matched to task demands, output quality, and system limitations rather than being indiscriminately high or low (Lee and See, 2004; Lelescu et al., 2025). Collaborative regulation refers to the active behavioral management of human AI interaction through prompting, checking, comparing, rewriting, and integrating (Weng et al., 2024; Lee et al., 2024). Perceived transfer gains refer to students' subjective perceptions that collaboration with GenAI has improved their evaluative judgment, reflective capacity, and ability to transfer strategies across tasks (Darvishi et al., 2024; Zhou et al., 2024).

This model contributes to the literature in three ways. It specifies more clearly how research centered on adoption, AI literacy, ethics, and learning outcomes can be linked within one explanatory chain rather than treated as separate literatures. It argues that trust calibration is not interchangeable with trust in general: its theoretical role is to connect contextual cues to students' later regulatory choices. It deliberately treats the outcome variable as perceived transfer gains rather than objective competence improvement, thereby aligning the claims of the study with the limits of cross sectional self report evidence.

Accordingly, the study asks the following questions:

- RQ1: What dimensions characterize university students' developmental experiences when collaborating with GenAI?
- RQ2: Do contextual support, trust calibration, collaborative regulation, and perceived transfer gains form structural relationships that are consistent with the proposed model?
- RQ3: Do trust calibration and collaborative regulation mediate the relationship between contextual support and perceived transfer gains?

## 2 Literature review and hypotheses

### 2.1 From separate literature streams to an integrated process account

Research on GenAI in higher education can be organized into four prominent literature streams, but these streams address different layers of the same educational process rather than four independent problems (Baig and Yadegaridehkordi, 2024). Adoption and institutional-integration research explains whether students and educators are willing and able to use GenAI and how perceived usefulness, social expectations, policy clarity, and course conditions shape its implementation (Almogren et al., 2024; Venkatesh et al., 2003; O'Dea, 2024; Kurtz et al., 2024). AI-literacy research specifies the knowledge and competencies needed for responsible use, including operational understanding, critical evaluation, ethical awareness, and recognition of system limitations (Ng et al., 2021; Chen et al., 2025; Jin et al., 2025; Anders and Speltz, 2025). Human–AI collaboration studies describe what students do with GenAI during writing, problem solving, and knowledge construction, revealing patterns that range from idea stimulation and iterative revision to passive dependence (Jeon and Lee, 2023; Nguyen et al., 2024; Atchley et al., 2024). Trust and self-regulated learning research, in turn, examines how students judge system reliability, monitor their own interaction, and decide whether to verify, revise, or rely on AI-generated material (Lee and See, 2004; Weng et al., 2024; Lee et al., 2024; Nazaretsky et al., 2025; Mart́ın-Moncunill and Alonso Mart́ınez, 2025). Viewed together, these literatures suggest a layered explanatory structure. Adoption and institutional integration describe the contextual conditions under which GenAI use becomes possible and normatively organized (Beckman et al., 2025). AI literacy identifies capacities that may support informed judgment, but possessing relevant knowledge does not guarantee that students will enact it during a particular task. Trust research addresses this judgment-level transition by explaining how users determine whether reliance is warranted. Human–AI collaboration and self-regulated learning research then address the behavioral level by showing how judgments are translated into prompting, monitoring, comparison, verification, and revision (Flink et al., 2025). Studies of student agency, critical thinking, and knowledge construction point to possible developmental outcomes of these interaction processes (Darvishi et al., 2024; Lee et al., 2024; Zhou et al., 2024). The four streams are therefore complementary: each explains a different part of the process through which access to GenAI may, or may not, become higher-quality learning activity. Social Cognitive Theory provides a broad organizing framework for this integration. It conceptualizes learning and action as emerging from reciprocal relations among environmental conditions, personal cognitive factors, and behavior (Schunk and DiBenedetto, 2020; Zimmerman, 2000). In the present model, contextual support represents the instructional and social environment surrounding GenAI use; trust calibration represents a task-sensitive cognitive appraisal; and collaborative regulation represents the behavioral management of interaction. Perceived transfer gains capture students' reported developmental interpretation of repeated collaboration (Jiang and Chen, 2026). This mapping does not imply that the variables operate in a permanently one-way sequence. Rather, it locates them at analytically different levels within a system in which environmental cues, judgments, and actions may influence one another over time. Self-Regulated Learning Theory provides a more proximal account of the judgment and action processes within this system. Social-cognitive models of self-regulation describe learning as a cycle involving task appraisal and planning, performance monitoring and control, and subsequent reflection (Zimmerman, 2000). Applied to GenAI collaboration, trust calibration captures part of the appraisal process through which students judge task risk, output reliability, and appropriate reliance. Collaborative regulation captures enacted control through iterative prompting, checking, comparison, rewriting, and selective integration. The outcomes of those actions may then inform later trust judgments, making the relationship analytically distinguishable but potentially reciprocal (Weng et al., 2024; Lee et al., 2024; Anders and Speltz, 2025; Qi et al., 2025). Cognitive Load Theory offers a complementary resource-allocation rationale. GenAI may reduce the effort required for routine idea generation or language production, but it can also create additional demands associated with evaluating uncertain claims, comparing sources, and deciding what should be retained or revised (Sweller, 1988; Darvishi et al., 2024). Because students cannot devote equal attention to every element of every AI response, task-sensitive trust judgments may help direct limited monitoring resources toward outputs for which error costs are higher, such as citations, factual claims, calculations, or disciplinary interpretations. Collaborative regulation then represents the actions through which those resources are deployed. Cognitive load is not measured in the present study, so this perspective is used to clarify why selective rather than indiscriminate verification may be educationally important, not as an additional tested mechanism. Human–automation interaction research provides the most direct theoretical basis for trust calibration (Lent, 2025). From this perspective, the goal is not to maximize trust but to support appropriate reliance by aligning users' trust with system capabilities, task conditions, and available evidence (Lee and See, 2004). In educational GenAI use, this means that reliance may be reasonable for one function, such as brainstorming, but require greater caution for another, such as generating evidence or interpreting disciplinary knowledge. This perspective also connects judgment to interaction: trust matters educationally because it influences whether students accept an output, seek additional evidence, request alternatives, or retain control over the final decision. This synthesis identifies a missing middle in the existing literature (Park, 2025). Adoption research explains whether GenAI is used, and outcome research describes what students may gain, but neither alone specifies how educational contexts are translated into learning-relevant activity. AI literacy identifies important competencies, but it does not by itself show whether those competencies are enacted in a given interaction. The present study therefore integrates these streams through a focused process account in which contextual support is related to trust calibration, trust calibration is related to collaborative regulation, and collaborative regulation is related to perceived transfer gains. The model does not claim to exhaust all reciprocal or cognitive mechanisms involved in GenAI-supported learning; it tests a theoretically specified set of relationships linking context, judgment, process, and subjective outcome.

### 2.2 Why trust calibration matters in educational use of GenAI

Within the integrated account developed above, trust calibration lies at the intersection of human–automation trust and self-regulatory task appraisal. The human–automation perspective explains why trust should be aligned with system capabilities and task conditions, whereas the self-regulated learning perspective explains how such judgments can guide monitoring and strategy selection and can subsequently be updated through feedback from action (Lee and See, 2004; Zimmerman, 2000). Trust has become a central topic in educational AI because students increasingly rely on systems that appear fluent, responsive, and socially conversational (Portocarrero Ramos et al., 2025). However, the educational problem is not simply whether students trust AI, but whether their reliance is appropriate to the task and available evidence. Recent work has shown that trust is multidimensional and context dependent in AI supported learning (Nazaretsky et al., 2025; Lelescu et al., 2025). Blind trust can encourage over reliance, whereas indiscriminate distrust can prevent learners from benefiting from useful scaffolding. A task sensitive concept is therefore needed. Trust calibration provides such a concept. Building on research in automation, trust calibration refers to the degree of fit between the user's trust and the system's actual capabilities in a given task context (Lee and See, 2004). In educational settings, this means that a student may reasonably rely on GenAI for brainstorming or language polishing, while remaining cautious about factual claims, citations, or disciplinary interpretation. Recent student-focused evidence further indicates that students' trust in AI is closely connected to the verification strategies they adopt, while verification practices remain uneven across learners (Mart́ın-Moncunill and Alonso Mart́ınez, 2025). This makes trust calibration theoretically useful because it captures a judgment process rather than a global attitude.

### 2.3 Construct boundaries: context, judgment, process, and outcome

The construct definitions used in the present study are integrative, theory-informed formulations rather than verbatim definitions adopted from a single prior source. Contextual support synthesizes research on instructional guidance, assessment design, peer norms, and institutional policy surrounding GenAI use in higher education (O'Dea, 2024; Kurtz et al., 2024). Trust calibration is adapted from the broader literature on trust in automation and appropriate reliance and is contextualized here as a task-sensitive judgment in educational GenAI use (Lee and See, 2004; Nazaretsky et al., 2025; Lelescu et al., 2025; Mart́ın-Moncunill and Alonso Mart́ınez, 2025). Collaborative regulation integrates self-regulated learning theory with emerging evidence on active management of human–AI interaction (Zimmerman, 2000; Nguyen et al., 2024; Weng et al., 2024; Lee et al., 2024; Zhou et al., 2024). Perceived transfer gains is an author-developed outcome label generated from the qualitative phase of this study and informed by related research on student agency, higher-order thinking, self-regulation, and critical evaluation (Darvishi et al., 2024; Lee et al., 2024; Zhou et al., 2024; Anders and Speltz, 2025). Study 1 subsequently refined these theoretical starting points by identifying how students described the four dimensions in concrete GenAI-supported learning experiences. A core concern in this study is the potential overlap among trust calibration, collaborative regulation, and perceived transfer gains. To reduce construct ambiguity, the model differentiates the four variables by analytic level. Contextual support is a context level construct: it describes the external instructional and social conditions that frame how GenAI may be used. Trust calibration is a judgment level construct: it refers to students' evaluative positioning toward AI in relation to task demands and system boundaries (Sarmurzin et al., 2026). Collaborative regulation is a process level construct: it refers to what students actually do during interaction, such as probing, verifying, comparing outputs, and revising drafts. Perceived transfer gains are an outcome level construct: they refer to what students report having developed as a consequence of repeated collaboration with GenAI. The distinction is important because constructs that are psychologically adjacent are not conceptually identical. Trust calibration is not the same as collaborative regulation: the former concerns whether and when an AI output should be treated as tentatively usable, whereas the latter concerns the actions taken after that judgment. Likewise, perceived transfer gains are not the same as trust calibration: students may report becoming better at judging information across tasks, but that retrospective outcome judgment still differs from the in task trust judgments that precede collaborative actions (Thomson et al., 2024). These construct boundaries are analytic rather than claims of strict temporal separation. Within a particular interaction episode, a student's judgment of output reliability and task risk may provide a proximal basis for deciding whether to accept, verify, compare, or revise an AI response. Across repeated exchanges, however, the outcomes of those regulatory actions can provide new evidence that updates subsequent trust judgments. Trust calibration and collaborative regulation may therefore form a recursive feedback loop at the broader activity level, consistent with cyclical accounts of self-regulated learning (Zimmerman, 2000; Anders and Speltz, 2025; Qi et al., 2025). The present study separates them conceptually in order to examine whether variation in judgment-level calibration is associated with enacted process control, not to suggest that the two processes are temporally isolated or exclusively unidirectional. Table 1 summarizes these distinctions.

**Table 1:** Construct definitions, theoretical origins, and boundaries.

Construct	Definition and theoretical role	Illustrative manifestation	What it is not
Contextual support	External instructional and social conditions that guide how GenAI may be used, including teacher guidance, assessment design, peer norms, and institutional policy; a context-level antecedent synthesized from research on GenAI integration in higher education (O'Dea, 2024; Kurtz et al., 2024).	Teacher guidance about permissible AI use, process-oriented assessment, peer modeling of responsible practice, and clear institutional rules.	Not a personal attitude or student behavior; it describes the environment that frames students' decisions.
Trust calibration	A task-sensitive judgment about when GenAI should be relied on, doubted, or verified; a judgment-level hinge construct adapted from research on trust calibration, appropriate reliance, and educational AI trust (Lee and See, 2004; Nazaretsky et al., 2025; Lelescu et al., 2025; Mart́ın-Moncunill and Alonso Mart́ınez, 2025).	Distinguishing between using GenAI for brainstorming and independently verifying factual claims, citations, or disciplinary interpretations.	Not the same as trust in AI in general, and not equivalent to verification behavior itself.
Collaborative regulation	The active management of human–AI interaction through iterative prompting, verification, comparison, rewriting, and selective integration; a process-level mechanism informed by self-regulated learning and human–AI collaboration research (Zimmerman, 2000; Nguyen et al., 2024; Weng et al., 2024; Lee et al., 2024; Zhou et al., 2024).	Iterative prompting, checking external sources, comparing outputs, rewriting generated text, and integrating AI output selectively.	Not a belief about AI quality; it concerns enacted process control during collaboration.
Perceived transfer gains	An author-developed outcome construct referring to students' perceptions that repeated GenAI collaboration has strengthened evaluative judgment, reflection, and strategy use across tasks; generated from Study 1 and informed by related research on agency, higher-order thinking, and self-regulated learning (Darvishi et al., 2024; Lee et al., 2024; Zhou et al., 2024; Anders and Speltz, 2025).	Reporting improved ability to assess information quality, reflect on weak reasoning, or reuse effective collaboration strategies in new academic tasks.	Not objective ability gain or evidence that competence improvement or causal transfer has been established.

These are integrative, theory-informed definitions developed for this study rather than verbatim definitions from a single source. Citations indicate their theoretical lineage. The first three constructs were synthesized from prior research and refined in Study 1; perceived transfer gains was labeled and operationalized from the qualitative findings and related learning research.

### 2.4 Contextual support, trust calibration, and collaborative regulation

From a social cognitive perspective, students' GenAI-related judgments and actions are situated within reciprocal relations among environmental conditions, personal cognition, and behavior (Schunk and DiBenedetto, 2020; Zimmerman, 2000). Contextual support represents the environmental component of this system: teacher guidance, assessment requirements, peer modeling, and institutional policy provide cues that can shape both students' judgments of appropriate reliance and the regulatory strategies they enact. In higher education, students do not encounter GenAI as isolated users. They encounter it within courses, assignments, classroom norms, peer discussions, and institutional policy. Research on GenAI integration repeatedly shows that guidance, assessment structure, and policy clarity shape whether students use AI superficially or responsibly (Kurtz et al., 2024; O'Dea, 2024; Saleh and ElSayary, 2025). Therefore:

- H1: Contextual support positively predicts trust calibration.

Supportive environments may also encourage students to regulate collaboration more actively. Process oriented assignments, requests for justification, and peer discussion norms make passive copying less viable and reflective engagement more likely. Therefore:

- H2: Contextual support positively predicts collaborative regulation.

### 2.5 Trust calibration as a bridge to collaborative regulation and perceived gains

Trust calibration should be associated with collaborative regulation, although the proposed direction should not be interpreted as denying reciprocity between the two processes. Within a particular decision episode, an evaluative judgment about the reliability of an AI response and the risk associated with the task provides one proximal basis for whether a student accepts the output, checks it against other evidence, compares alternatives, or rewrites it. Students whose trust is more sensitive to task demands and system limitations should therefore be more likely to maintain active control over the interaction rather than delegate the task wholesale to AI. Research on AI-assisted writing and self-regulated learning similarly associates productive GenAI use with iterative prompting, critical inspection, and revision rather than one-shot answer acceptance (Nguyen et al., 2024; Weng et al., 2024; Lee et al., 2024). At the same time, self-regulated learning is cyclical rather than strictly linear (Zimmerman, 2000; Anders and Speltz, 2025; Qi et al., 2025). Verification outcomes, detected errors, unsuccessful prompts, and effective revisions may all feed back into students' subsequent judgments about when and how much GenAI should be trusted. The path from trust calibration to collaborative regulation therefore represents the focal analytic direction tested in the present model, while a reverse feedback pathway may operate across later prompts or task episodes.

Therefore:

- H3: Trust calibration is positively associated with collaborative regulation in the proposed structural model.

Trust calibration may also have a direct association with perceived developmental outcomes. When students learn to align reliance with task conditions, they may perceive stronger gains in judgment and reflective capacity across tasks. This would be consistent with educational trust research showing that trust shapes willingness to use, perceived usefulness, and patterns of reliance (Nazaretsky et al., 2025; Lelescu et al., 2025). Therefore:

- H4: Trust calibration positively predicts perceived transfer gains.

### 2.6 Collaborative regulation and perceived transfer gains

The process most likely to connect GenAI use with developmental outcomes is collaborative regulation. Recent studies have shown that guided or self regulated uses of GenAI are more likely to support critical thinking, learning strategy development, and knowledge construction than unstructured answer retrieval (Darvishi et al., 2024; Lee et al., 2024; Zhou et al., 2024). If students repeatedly engage in probing, checking, comparing, and rewriting, they are more likely to perceive gains that extend beyond the immediate task. Therefore:

- H5: Collaborative regulation positively predicts perceived transfer gains.

### 2.7 Indirect pathways

Taken together, the tested model specifies several directional indirect pathways. Contextual support is expected to be associated with perceived transfer gains indirectly through trust calibration. Trust calibration is expected to be associated with perceived transfer gains indirectly through collaborative regulation. The model tests an analytically ordered pathway in which contextual support is linked to trust calibration, trust calibration is linked to collaborative regulation, and collaborative regulation is linked to perceived transfer gains. This ordering represents the focal direction examined in the cross-sectional structural model rather than a claim that trust judgment and regulatory action form a permanently one-way sequence. Across repeated interactions, collaborative regulation may also provide feedback that recalibrates later trust judgments.

- H6: Trust calibration mediates the relationship between contextual support and perceived transfer gains.
- H7: Collaborative regulation mediates the relationship between trust calibration and perceived transfer gains.
- H8: Contextual support has a positive chained indirect effect on perceived transfer gains through trust calibration and collaborative regulation.

Figure 1 presents the conceptual model.

**Figure 1:**
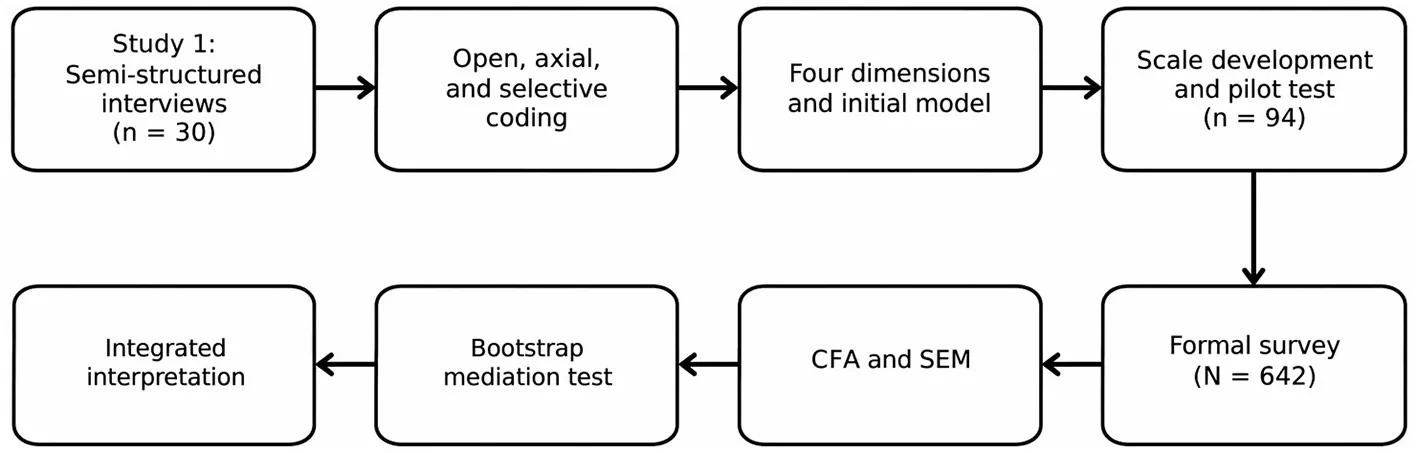
Conceptual model.

## 3 Method

### 3.1 Design, philosophical stance, and integration logic

The study used an exploratory sequential mixed methods design and was guided by a pragmatic philosophical stance (Robinson, 2007). Pragmatism was appropriate because the research problem required both an interpretive understanding of how students described their collaboration with GenAI and an empirical examination of whether the resulting constructs formed relationships consistent with the proposed model. Rather than privileging either qualitative or quantitative evidence as inherently superior, the pragmatic stance placed the research questions at the center of methodological decision making and treated different forms of evidence as complementary resources for addressing different aspects of the problem. The two phases therefore had distinct but connected epistemic roles. Study 1 used semi-structured interviews to generate contextually grounded categories, clarify construct boundaries, and identify how students described contextual support, trust calibration, collaborative regulation, and perceived transfer gains in concrete academic experiences. Study 2 translated these qualitative categories into a self-report instrument and examined the measurement properties and structural relationships of the resulting constructs in a larger sample. The quantitative phase was not intended to replace the situated meanings generated in the qualitative phase; rather, it examined whether those meanings could be represented by a coherent measurement structure and whether their associations were consistent with the proposed theoretical account. Integration occurred at three points. Integration occurred at the design level because the findings of Study 1 determined the constructs and relationships examined in Study 2. Integration occurred at the methods level because the qualitative codes and categories directly informed item generation and construct operationalization. Integration occurred at the interpretation level because the structural results were interpreted alongside the interview evidence, particularly when explaining construct boundaries and the proposed relationships among context, judgment, regulation, and perceived outcome. This connecting logic is central to an exploratory sequential design rather than an incidental combination of two independent studies. The pragmatic stance also informed the limits placed on inference. The qualitative findings were treated as contextually situated interpretations, while the quantitative findings were treated as theory-consistent structural evidence rather than proof of temporal or causal mechanisms. Accordingly, the study does not assume that either phase provides a complete or final representation of students' collaboration with GenAI. Instead, the two phases jointly provide a practically useful but provisional account that can be refined through longitudinal, behavioral, experimental, and cross-cultural research.

### 3.2 Study 1: qualitative exploration

The qualitative phase recruited 38 Chinese undergraduates using purposeful maximum-variation sampling. Recruitment was conducted from September to November 2025 through course announcements, student learning communities, and instructor referrals at five mainland Chinese universities. The recruitment notice described the study as an investigation of university students' experiences of using GenAI for academic learning and invited interested students to complete a brief eligibility screening form. A total of 54 students initially expressed interest. Nine did not meet the eligibility criteria because their use of GenAI was occasional or they were unable to describe a concrete academic-use episode. Of the 45 eligible students, five were unable to participate because of scheduling conflicts or later withdrawal, and two interviews were canceled before data collection. The final qualitative sample therefore comprised 38 participants. Eligibility required participants to be currently enrolled undergraduates, to have used GenAI repeatedly for academic tasks during the preceding semester, and to be able to describe at least one specific episode of GenAI-supported learning. Students were excluded if their use was only occasional, if they could not provide task-specific examples, or if they were unwilling to provide informed consent or be audio recorded. Purposeful selection was used to obtain variation in year of study, disciplinary background, and reported level of GenAI use. Participants represented humanities, social sciences, business, and STEM-related majors across all four undergraduate years. Thirty-two interviews formed the main analytic sample. After the preliminary category structure had been established, six additional eligible participants were interviewed to examine whether further cases generated new core dimensions. These six interviews produced no new core categories and were therefore treated as saturation-check cases. Interviews were conducted online or face to face according to participant availability. Participants received a small reimbursement equivalent to RMB 30 for their time, and the researchers responsible for recruitment did not have a direct teaching or assessment relationship with the participants. Participant characteristics are summarized in Table 2.

**Table 2:** Characteristics of Study 1 participants.

Characteristic	Category	*n*	%
Sample role	Main analytic sample	32	84.2
	Saturation-check sample	6	15.8
Gender	Female	21	55.3
	Male	17	44.7
Age	Mean (SD)	20.4 (1.2)	
	Range	18–23 years	
Year of study	Year 1	8	21.1
	Year 2	11	28.9
	Year 3	12	31.6
	Year 4	7	18.4
Disciplinary area	Humanities	10	26.3
	Social sciences	8	21.1
	Business	7	18.4
	STEM-related disciplines	13	34.2
Interview mode	Online	23	60.5
	Face-to-face	15	39.5

Percentages are based on the full qualitative sample of 38 participants. The main analytic sample comprised 32 participants, while six additional participants were retained for theoretical saturation checking. All participants were undergraduates recruited from multiple mainland Chinese universities and had used GenAI for academic tasks on a sustained basis during the preceding semester.

The semi-structured interview protocol was developed through an iterative process linking the research questions, the initial literature review, and the exploratory purpose of Study 1. An initial protocol containing 11 questions was drafted to elicit concrete accounts of students' academic use of GenAI rather than general attitudes toward the technology. The questions were organized around six domains: initial motivation, collaborative practices, perceived benefits and challenges, risk and reliability judgments, contextual influences, and perceived development. These domains were intended to capture external conditions, task-related judgments, interaction processes, and perceived outcomes without requiring participants to use the theoretical terminology later adopted in the model. The draft protocol was reviewed by two researchers in educational psychology and two higher education instructors. The reviewers evaluated the relevance of each question to the research objectives, wording clarity, neutrality, redundancy, and the likelihood that the question would elicit a task-specific example. They were also asked to identify questions that might presuppose positive effects of GenAI or lead participants toward a preferred response. On the basis of their comments, two overlapping questions concerning learning benefits were combined, three questions were simplified to reduce technical wording, and additional probes were added to elicit unsuccessful, risky, or contradictory experiences with GenAI. Four questions were converted into follow-up probes, and two overlapping questions were combined, resulting in six core questions and eight optional probes. The revised protocol was piloted with four undergraduate students who met the general eligibility criteria but were not included in the main qualitative sample. After each pilot interview, participants were asked whether any question was unclear, repetitive, difficult to answer, or suggestive of a preferred response. The research team also assessed whether the questions generated sufficiently detailed accounts of specific tasks, judgments, and actions. The pilot indicated that participants initially tended to provide broad evaluations of GenAI rather than detailed examples. Consequently, the order of two questions was adjusted, the wording of three follow-up prompts was revised, and probes such as “Can you describe a specific occasion?” and “What did you do after receiving that response?” were added. Table 3 presents the six finalized core questions. During the formal interviews, the interviewer used follow-up prompts flexibly, including “What made you trust or question that response?”, “How did you decide what to retain?”, and “Did this experience affect how you approached a later task?” These probes were used to clarify the sequence and meaning of participants' experiences rather than to impose the later construct labels. Each interview lasted approximately 45–70 min. All interviews were audio recorded with informed consent, transcribed verbatim, checked against the recordings for completeness, and anonymized before analysis. The four pilot interviews were used only to refine the protocol and were not included in the coded corpus.

**Table 3:** Core interview prompts.

Prompt	Question focus
Q1	Why did you first start using GenAI in your academic work?
Q2	How do you usually collaborate with GenAI in coursework, writing, or everyday study tasks?
Q3	What benefits or difficulties has GenAI created for your learning and thinking?
Q4	How do you judge whether AI output is trustworthy, risky, or in need of verification?
Q5	How do teachers, peers, course requirements, and platform rules influence your AI use?
Q6	After repeated use, what kinds of abilities do you think you have developed, or hope to develop, through collaboration with AI?

The transcripts were analyzed using open, axial, and selective coding. In the first round, the team segmented the material into meaning units and generated initial codes. In the second round, related codes were grouped into higher order categories. In the third round, the categories were integrated into a process model centered on the developmental experience of collaborating with GenAI in higher education. The coding process yielded 286 meaning units, 44 initial codes, and 11 axial categories, which were then integrated into the four focal constructs. Two coders first worked independently on a randomly selected subset corresponding to roughly one quarter of the transcripts and then met to reconcile discrepancies, revise the codebook, and document decision rules. Agreement on the calibration subset reached Cohen's κ = 0.85. The remaining transcripts were then coded using the revised codebook, with disagreements discussed until consensus was achieved. The additional six cases did not generate new core dimensions, which was taken as evidence of category saturation. The coding team consisted of researchers trained in educational psychology and higher education studies. To reduce premature confirmation of the proposed model, the team used analytic memos, iterative codebook revision, and constant comparison across cases before moving to selective coding.

Table 4 shows how the qualitative material was transformed analytically, linking verbatim interview excerpts to open codes, axial categories, and the four core dimensions.

**Table 4:** Illustrative verbatim excerpts from the coded corpus and analytic progression.

Illustrative verbatim excerpt	Open code	Axial category	Core dimension
“My instructor said AI can help with brainstorming, but the final argument and evidence still have to be mine.”	Boundary cue from instruction	Course and assessment guidance	Contextual support
“When my classmates compare the answer with papers or class notes, it feels normal that I should check it too.”	Peer modeling of verification	Social norm for responsible use	Contextual support
“I can use it to generate ideas or polish wording, but if it gives a citation or a technical claim, I will not trust it until I verify it.”	Task dependent reliance	Judgment of appropriate reliance	Trust calibration
“Sometimes the answer sounds so confident that you want to believe it, and that is exactly when I remind myself to slow down and check.”	Fluency risk awareness	Recognition of system limitation	Trust calibration
“I usually ask a second or third question, or ask for another version, before I decide what to keep.”	Iterative prompting and comparison	Active interaction control	Collaborative regulation
“I rarely paste it directly. I rewrite it so it matches my own structure and what the course expects.”	Transformative revision	Ownership preserving regulation	Collaborative regulation
“After checking AI so many times, I notice weak claims faster even when I am reading articles or classmates' drafts.”	Extended evaluative awareness	Cross task judgment benefit	Perceived transfer gains
“Now, even in assignments where I do not use AI much, I still break the task apart, compare versions, and ask what evidence supports each point.”	Strategy reuse across tasks	Perceived transfer of learning strategy	Perceived transfer gains

The qualitative phase did not simply provide narrative context for a pre existing model. It directly informed scale development. The four core dimensions were translated into an initial 25 item pool, and the hypothesized paths were only finalized after the dimensions had stabilized analytically.

### 3.3 Study 2: quantitative validation

Instrument development followed the sequential logic of the mixed methods design (Table 5). The survey was not assembled solely from pre-existing scales. Instead, the qualitative findings provided the primary content basis, while relevant literature was used to clarify construct boundaries and refine item wording. The coding process generated 286 meaning units, 44 initial codes, and 11 axial categories, which were integrated into four core dimensions: contextual support, trust calibration, collaborative regulation, and perceived transfer gains. The research team reviewed the illustrative quotations, open codes, axial categories, and construct definitions together and drafted items representing recurring and conceptually central manifestations of each dimension. Several principles guided initial item writing. Each item was designed to represent one construct and one primary idea; double-barreled statements, technical terminology, and overly general evaluations of GenAI were avoided. Contextual-support items focused on external instructional and social conditions. Trust-calibration items focused on task-sensitive judgments of appropriate reliance rather than verification behavior itself. Collaborative-regulation items focused on enacted prompting, checking, comparison, rewriting, and selective integration. Perceived-transfer-gain items focused on subjective perceptions of developmental change across tasks rather than objectively demonstrated competence. This process produced an initial pool of 25 items: seven items for contextual support, six for trust calibration, seven for collaborative regulation, and five for perceived transfer gains. All items were rated on a five-point Likert scale ranging from 1 (*strongly disagree*) to 5 (*strongly agree*). The initial item pool was reviewed independently by two researchers in educational psychology and two higher education instructors. Reviewers assessed construct relevance, coverage of the qualitative categories, wording clarity, suitability for undergraduate respondents, and potential overlap among the four dimensions. They classified each item as “retain,” “revise,” or “remove” and provided written comments where revision was recommended. Across the four reviews, 15 items were judged suitable without substantive changes, eight required minor wording revisions, and two required substantial revision because their wording overlapped with more than one construct. The research team discussed all comments and reached consensus on the revised wording. No item was removed at this stage because all four constructs retained meaningful content coverage, and the revised instrument therefore continued to contain 25 items. This procedure was used as a qualitative assessment of content validity; no numerical content-validity index was calculated. The revised instrument was pilot tested with an independent sample of 94 undergraduate students recruited from two universities that did not contribute participants to the formal survey sample. The pilot participants completed the questionnaire anonymously online. Their mean completion time was 8.7 min, and fewer than 2% of item responses were missing. Preliminary inspection showed that all response categories were used and that no item exhibited severe floor or ceiling effects. Exploratory factor analysis used principal axis factoring with oblimin rotation because the constructs were expected to correlate. The Kaiser-Meyer-Olkin value was 0.91, and Bartlett's test of sphericity was statistically significant, χ^2^(300) = 1458.62, *p* < 0.001, indicating that the item correlation matrix was suitable for factor analysis. Factor retention was informed jointly by parallel analysis, eigenvalues greater than 1, inspection of the scree plot, and conceptual interpretability. Items were retained when they had a primary loading of at least 0.50, communality above 0.40, corrected item–total correlation above 0.40, and no problematic secondary loading above 0.30 or loading difference smaller than 0.20. The analyses supported a four-factor solution. Three items were removed. One contextual-support item had a weak primary loading of 0.43 and a communality of 0.36. One trust-calibration item loaded on both trust calibration and collaborative regulation (0.52 and 0.39, respectively), producing an insufficient loading difference of 0.13. One collaborative-regulation item had a corrected item–total correlation of 0.34 and substantially duplicated the content of a more clearly worded item. After their removal, the retained item loadings ranged from 0.58 to 0.83, and the four factors remained conceptually interpretable. The remaining items were re-examined to ensure that each construct retained adequate conceptual coverage. Minor wording changes were made to four items to improve consistency and readability before formal administration. The final instrument contained 22 items: six measuring contextual support, five measuring trust calibration, six measuring collaborative regulation, and five measuring perceived transfer gains. Table 6 summarizes the pilot-stage decision rules and outcomes.

**Table 5:** How the qualitative phase informed the quantitative phase.

Core dimension	Qualitative insight translated into items	Related structural role
Contextual support	Items captured teacher guidance, process oriented assessment, peer modeling, and institutional clarity regarding acceptable AI use.	Exogenous antecedent
Trust calibration	Items captured task dependent trust, awareness of AI limitations, and resistance to taking fluent output as automatically correct.	Judgment level mediator
Collaborative regulation	Items captured iterative prompting, verification, comparison, rewriting, and integration practices during human AI interaction.	Process level mediator
Perceived transfer gains	Items captured students' self reported improvement in evaluating information, reflecting on output quality, and transferring collaboration strategies across tasks.	Outcome variable

**Table 6:** Pilot stage scale development criteria and outcomes.

Decision domain	Criterion	Observed outcome
Factor extraction	Principal axis factoring with oblimin rotation	Four factor solution retained
Factor retention	Parallel analysis, scree plot, and eigenvalues >1.00	Four factors converged conceptually and statistically
Item quality	Primary loading ≥0.50; communality ≥0.40; corrected item total correlation ≥0.40	22 items retained
Cross loading	Secondary loading < 0.30 or loading gap ≥0.20	3 items removed for weak or ambiguous loadings
Content validity	Expert review for construct alignment and wording clarity	Minor wording revisions before formal administration

The formal survey distributed 680 questionnaires and received 654 returns. Responses were excluded when completion times were abnormally short, long string patterns suggested inattentive responding, missing data exceeded 20%, or logic check items were failed. The final analytic sample contained 642 responses (effective rate = 94.41%). To reduce common method bias procedurally, the questionnaire emphasized anonymity, stated that there were no right or wrong answers, and distributed focal items across the survey rather than clustering them by construct.

Contextual support measured perceived instructional and social guidance for GenAI use. Trust calibration measured students' task sensitive judgments regarding when AI output should be trusted, doubted, or verified. Collaborative regulation measured enacted interaction strategies such as iterative prompting, fact checking, comparison, and rewriting. Perceived transfer gains measured subjective perceptions of strengthened evaluative judgment, reflection, and cross task strategy use.

The quantitative analysis began with response-quality screening. Questionnaires were excluded when completion times were shorter than one third of the sample median, when pronounced long-string response patterns were detected, when embedded logic-check items were failed, or when more than 20% of the responses were missing. The retained cases contained complete data on all 22 focal items and the four control variables; therefore, no item-level imputation or model-based missing-data treatment was required. The distributions of the 22 observed items and the four focal constructs were examined using means, standard deviations, skewness, and kurtosis. Multivariate normality was assessed using Mardia's multivariate skewness and kurtosis statistics. Because the data did not satisfy strict multivariate normality, confirmatory factor analysis (CFA) and structural equation modeling (SEM) were estimated using robust maximum likelihood with robust standard errors and a scaled chi-square statistic. Descriptive statistics and zero-order correlations were calculated for the four focal constructs. CFA was then conducted to evaluate the hypothesized four-factor measurement model. Model fit was assessed using the chi-square statistic, the comparative fit index (CFI), the Tucker–Lewis index (TLI), the root mean square error of approximation (RMSEA), and the standardized root mean square residual (SRMR). Internal consistency and convergent validity were evaluated using Cronbach's alpha, composite reliability (CR), and average variance extracted (AVE). Discriminant validity was assessed using the Fornell–Larcker criterion and heterotrait–monotrait (HTMT) ratios. Measurement invariance was examined across gender and disciplinary-area groups. Configural invariance was tested first, followed by metric invariance through equality constraints on factor loadings and scalar invariance through additional equality constraints on item intercepts. Invariance was evaluated primarily using changes in CFI, RMSEA, and SRMR because chi-square difference tests are sensitive to sample size. Metric invariance was considered supported when |ΔCFI| ≤ 0.010, ΔRMSEA ≤ 0.015, and ΔSRMR ≤ 0.030. For scalar invariance, the corresponding SRMR criterion was 0.010 (Chen, 2007). Potential common method bias was examined through the procedural remedies incorporated into questionnaire administration and Harman's single-factor test. Non-response bias was assessed indirectly by comparing early and late respondents on demographic characteristics and the four focal constructs (Struminskaya and Gummer, 2022). Because individual-level information was unavailable for students who did not return the questionnaire, the early–late comparison was treated as a proxy rather than a definitive test of non-response bias. SEM was used to test the hypothesized structural relationships while controlling for gender, year of study, disciplinary area, and weekly GenAI use. Indirect effects were estimated using 5,000 bootstrap resamples, with statistical significance determined from 95% bootstrap confidence intervals. The magnitude of the structural relationships was evaluated using standardized path coefficients, standardized indirect effects, and the proportion of explained variance (*R*^2^) in each endogenous construct. Variance inflation factors were examined to assess multicollinearity, and a sensitivity analysis excluding cases with comparatively high leverage was conducted to determine whether the focal structural estimates were disproportionately influenced by a small number of observations.

Figure 2 summarizes the overall research process.

**Figure 2:**
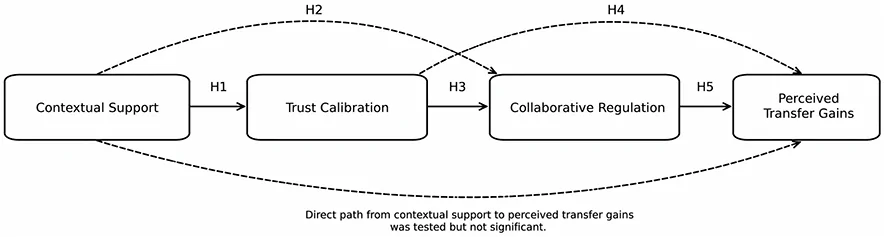
Research process of the exploratory sequential mixed methods design.

#### 3.3.1 Ethics, transparency, and data availability

Participants provided informed consent before participation, and all interview and survey materials were anonymized prior to analysis. The study followed the institutional procedures governing human participant research and data protection in university based educational research.

## 4 Results

### 4.1 Qualitative findings

The qualitative phase supported an analytically differentiated and potentially recursive interpretation of students' collaboration with GenAI. Rather than treating the four dimensions as interchangeable aspects of positive AI use, participants' accounts differentiated external contextual cues, task-sensitive trust judgments, enacted regulation during interaction, and perceived developmental outcomes across tasks. This organization corresponds to the construct boundaries summarized in Table 1.

Participants described contextual support primarily through explicit instructional boundaries, assessment expectations, and peer norms. One participant explained, “My instructor said AI can help with brainstorming, but the final argument and evidence still have to be mine.” Another added, “When my classmates compare the answer with papers or class notes, it feels normal that I should check it too.” The first account illustrates how instructor guidance defines the permitted role of GenAI while preserving students' responsibility for argumentation and evidence. The second shows how peer practices normalize verification as an expected academic behavior. Consistent with the definition in Table 1, these examples locate contextual support in the external instructional and social environment rather than in students' personal attitudes or regulatory actions. The context provides boundary cues and normative expectations that can frame later judgments about when AI output is appropriate to use.

Trust calibration appeared in participants' efforts to adjust reliance according to task type, output risk, and perceived system limitations. As one student put it, “I can use it to generate ideas or polish wording, but if it gives a citation or a technical claim, I will not trust it until I verify it.” Another noted, “Sometimes the answer sounds so confident that you want to believe it, and that is exactly when I remind myself to slow down and check.” The first account directly illustrates task-sensitive judgment: the participant regarded GenAI as relatively usable for brainstorming and language support but treated factual, evidential, and technical claims as requiring greater caution. The second account shows awareness that fluent presentation can create an unwarranted impression of reliability. Together, these statements support the definition of trust calibration as adjusting reliance to task demands and system limitations rather than maintaining uniformly high or low trust. They also reinforce the construct boundary identified in Table 1: the focal phenomenon is the judgment that an output should be treated cautiously or verified, whereas the enacted checking, comparison, or revision is coded as collaborative regulation. This distinction is analytic rather than strictly chronological. Once students check an output, discover an error, or observe that a revision improves the result, that experience may feed back into how they calibrate trust in the next prompt or task.

Collaborative regulation was evident in descriptions of active process control during human–AI interaction. One participant said, “I usually ask a second or third question, or ask for another version, before I decide what to keep.” Another explained, “I rarely paste it directly. I rewrite it so it matches my own structure and what the course expects.” The first account reflects iterative prompting and comparison rather than acceptance of the initial output. The second demonstrates transformative revision and selective integration, through which the student retains ownership of the final academic product. These practices go beyond recognizing that AI may be unreliable; they represent observable actions taken to manage the interaction. This distinction supports the process-level definition in Table 1, according to which collaborative regulation concerns enacted prompting, checking, comparing, rewriting, and integration rather than a belief about AI quality.

Perceived transfer gains were expressed as reported changes extending beyond the immediate GenAI-supported task. One student reflected, “After checking AI so many times, I notice weak claims faster even when I am reading articles or classmates' drafts.” Another reported, “Now, even in assignments where I do not use AI much, I still break the task apart, compare versions, and ask what evidence supports each point.” These accounts suggest perceived carryover in two forms. The first concerns evaluative judgment across other information sources, while the second concerns reuse of comparison and evidence-checking strategies in tasks where AI is not central. They therefore correspond to the cross-task judgment and strategy-transfer manifestations identified in Table 1. At the same time, the evidence remains retrospective and subjective: participants reported feeling more capable of identifying weak claims or reusing regulatory strategies, but the interviews did not independently test whether their objective competence had improved. Taken together, the qualitative findings clarify the boundaries and potentially recursive relations among the four constructs. Within particular decision episodes, task-sensitive judgments could guide checking, comparison, and revision, whereas the outcomes of those actions could inform subsequent trust judgments. The evidence is therefore consistent with the focal structural ordering but does not establish a rigid temporal sequence.

### 4.2 Data screening, distributional properties, and descriptive statistics

Of the 654 returned questionnaires, 12 were excluded before substantive analysis (Table 7). Five questionnaires had completion times shorter than one third of the sample median, four showed pronounced long-string response patterns, two failed the embedded logic-check items, and one contained more than 20% missing responses. The final analytic sample therefore contained 642 cases. All retained cases had complete data on the 22 focal items and the four control variables. Consequently, no item-level imputation or model-based missing-data treatment was required. The distributions of the observed items were examined before confirmatory and structural analyses. Across the 22 items, skewness values ranged from −0.81 to 0.16, and kurtosis values ranged from −0.68 to 0.91. For the four composite variables, skewness ranged from −0.46 to −0.12, and kurtosis ranged from −0.31 to 0.37. These values indicated no severe univariate departure from normality. However, Mardia's multivariate skewness coefficient was 31.46 (*p* < 0.001), and the multivariate kurtosis coefficient was 550.36, with a normalized estimate of 8.72 (*p* < 0.001). Thus, strict multivariate normality was not supported. Given the modest univariate deviations but statistically significant multivariate non-normality, the CFA and SEM were estimated using robust maximum likelihood with robust standard errors and a scaled chi-square statistic. Bootstrap confidence intervals based on 5,000 resamples were retained for the indirect-effect analyses. Table 8 presents the descriptive statistics and zero-order correlations for the four focal constructs. All focal correlations were positive and statistically significant. Contextual support correlated moderately with trust calibration and collaborative regulation. Collaborative regulation showed the strongest zero-order association with perceived transfer gains, consistent with its proposed role as the proximal process variable.

**Table 7:** Formal sample profile.

Sample characteristic	Category	Proportion/value
Gender	Female/male	56.1%/43.9%
Year of study	Year 1/year 2/year 3/year 4	21.5%/29.7%/31.8%/17.0%
Disciplinary area	Humanities/STEM/ business and others	34.6%/45.9%/19.5%
Weekly GenAI use	Average number of academic uses per week	4.8
Final analytic sample	Valid responses/returned responses	642/654

**Table 8:** Descriptive statistics and correlations.

Variable	M	SD	1	2	3	4
1. Contextual support	3.78	0.67				
2. Trust calibration	3.64	0.72	0.55***			
3. Collaborative regulation	3.71	0.69	0.48***	0.60***		
4. Perceived transfer gains	3.69	0.73	0.36***	0.48***	0.68***	

***p < 0.001.

### 4.3 Measurement model

The four factor CFA showed acceptable model fit, χ^2^/*df* = 2.34, CFI = 0.953, TLI = 0.946, RMSEA = 0.046, and SRMR = 0.042. Standardized factor loadings ranged from 0.66 to 0.84. Reliability and convergent validity were adequate: Cronbach's alpha values ranged from 0.841 to 0.886, CR values ranged from 0.84 to 0.90, and AVE values ranged from 0.57 to 0.65 (Table 9). Table 10 further indicates that the square root of each construct's AVE exceeded its inter construct correlations, and all HTMT ratios were below 0.85, supporting discriminant validity.

**Table 9:** Reliability and convergent validity.

Latent variable	Cronbach's α	CR	AVE
Contextual support	0.841	0.84	0.57
Trust calibration	0.868	0.88	0.61
Collaborative regulation	0.886	0.90	0.65
Perceived transfer gains	0.873	0.87	0.60
Overall scale	0.912	NA	NA

**Table 10:** Discriminant validity (Fornell Larcker matrix; square roots of AVE on the diagonal).

Construct	1	2	3	4
1. Contextual support	0.755			
2. Trust calibration	0.55	0.781		
3. Collaborative regulation	0.48	0.60	0.806	
4. Perceived transfer gains	0.36	0.48	0.68	0.775

HTMT ratios ranged from 0.58 to 0.83, below the conventional 0.85 threshold.

Multi-group CFA was conducted to examine whether the four-factor measurement model operated comparably across gender and disciplinary-area groups. The gender comparison included 360 female and 282 male participants. The disciplinary-area comparison included 222 humanities students, 295 STEM students, and 125 students from business and other disciplines.

As shown in Table 11, the unconstrained configural models showed acceptable fit for both grouping variables, indicating that the same four-factor structure was supported across groups. For gender, constraining the factor loadings to equality resulted in ΔCFI = −0.001, ΔRMSEA = −0.001, and ΔSRMR = 0.004. Adding equality constraints on item intercepts resulted in ΔCFI = −0.002, ΔRMSEA = 0.000, and ΔSRMR = 0.004.

**Table 11:** Measurement invariance across gender and disciplinary area.

Grouping variable	Model	χ^2^	df	CFI	RMSEA	SRMR	ΔCFI	ΔRMSEA	ΔSRMR
Gender	Configural	837.24	406	0.951	0.041	0.045	–	–	–
	Metric	858.91	424	0.950	0.040	0.049	−0.001	−0.001	0.004
	Scalar	885.37	442	0.948	0.040	0.053	−0.002	0.000	0.004
Disciplinary area	Configural	1284.63	609	0.947	0.042	.052	–	–	–
	Metric	1321.46	645	0.946	0.040	0.057	−0.001	−0.002	0.005
	Scalar	1369.88	681	0.944	0.040	0.061	−0.002	0.000	0.004

The gender comparison included 360 female and 282 male participants. The disciplinary-area comparison included 222 humanities students, 295 STEM students, and 125 students from business and other disciplines. Metric models constrained factor loadings to equality across groups. Scalar models additionally constrained item intercepts. Changes in fit indices are calculated relative to the immediately preceding model. CFI, comparative fit index; RMSEA, root mean square error of approximation; SRMR, standardized root mean square residual.

For disciplinary area, the metric model produced ΔCFI = −0.001, ΔRMSEA = −0.002, and ΔSRMR = 0.005. The scalar model produced ΔCFI = −0.002, ΔRMSEA = 0.000, and ΔSRMR = 0.004. All changes were within the recommended criteria for measurement invariance. The results therefore supported configural, metric, and scalar invariance across gender and disciplinary area, suggesting that the four constructs were measured sufficiently comparably across the tested groups.

### 4.4 Response quality, common method bias, and non-response bias

Response-quality screening was conducted before the measurement and structural analyses. Of the 654 returned questionnaires, five were excluded because their completion times were shorter than one third of the sample median, four showed pronounced long-string response patterns, two failed the embedded logic-check items, and one contained more than 20% missing data. The remaining 642 questionnaires contained complete data on the focal measures and control variables. Variance inflation factors ranged from 1.48 to 2.31, indicating no serious multicollinearity among the predictors. Sensitivity analyses excluding seven cases with comparatively high leverage did not materially change the direction, magnitude, or statistical significance of the focal structural paths. Because Study 2 relied on a single self-report questionnaire, common method bias remained a genuine concern. Procedural remedies included anonymous participation, neutral instructions, an explicit statement that there were no right or wrong answers, and distribution of the focal items across the questionnaire rather than presentation in four visibly separated construct blocks. Harman's single-factor test showed that the first unrotated factor accounted for 28.4% of the total variance, well below a majority of the explained variance. This result suggested that a single dominant factor was not present. Nevertheless, because no marker variable or latent method factor was included, Harman's test should be treated as a limited diagnostic rather than as evidence that common method effects were absent. The questionnaire was distributed to 680 students, of whom 654 returned a response, corresponding to an initial response rate of 96.18%. Non-response bias was assessed indirectly using a response-wave analysis. The earliest 25% of valid respondents (*n* = 161) were compared with the latest 25% (*n* = 161), with late respondents treated as a proxy for non-respondents. Chi-square tests showed no statistically significant early–late differences in gender, χ^2^(1) = 0.49, *p* = 0.484; year of study, χ^2^(3) = 1.82, *p* = 0.610; or disciplinary area, χ^2^(2) = 1.21, *p* = 0.547. Independent-samples tests also showed no statistically significant differences between early and late respondents in weekly GenAI use, *t*(320) = 0.92, *p* = 0.359, *d* = 0.10; contextual support, *t*(320) = 0.68, *p* = 0.498, *d* = 0.08; trust calibration, *t*(320) = 0.44, *p* = 0.660, *d* = 0.05; collaborative regulation, *t*(320) = 1.11, *p* = 0.268, *d* = 0.12; or perceived transfer gains, *t*(320) = 0.79, *p* = 0.431, *d* = 0.09. All standardized mean differences were small. These findings provided no clear evidence of systematic response-wave differences. However, because individual-level data were unavailable for the 26 students who did not return the questionnaire, this analysis cannot definitively rule out non-response bias.

### 4.5 Structural model and hypothesis testing

Table 12 presents the focal SEM results. Contextual support significantly predicted trust calibration (β = 0.56, *p* < 0.001), supporting H1, and collaborative regulation (β = 0.18, *p* = 0.004), supporting H2. Trust calibration significantly predicted collaborative regulation (β = 0.49, *p* < 0.001) and perceived transfer gains (β = 0.22, *p* < 0.001), supporting H3 and H4. Collaborative regulation significantly predicted perceived transfer gains (β = 0.54, *p* < 0.001), supporting H5. The direct path from contextual support to perceived transfer gains was not statistically significant (β = 0.07, *p* = 0.183). Among the control variables, weekly GenAI use showed a small positive association with collaborative regulation (β = 0.11, *p* = 0.021) and perceived transfer gains (β = 0.09, *p* = 0.041), whereas gender, year of study, and disciplinary area were not statistically significant in the final model.

**Table 12:** Structural model path estimates.

Path	Standardized β	*p*	Conclusion
Contextual support → Trust calibration	0.56	< 0.001	Supports H1
Contextual support → Collaborative regulation	0.18	0.004	Supports H2
Trust calibration → Collaborative regulation	0.49	< 0.001	Supports H3
Trust calibration → Perceived transfer gains	0.22	< 0.001	Supports H4
Collaborative regulation → Perceived transfer gains	0.54	< 0.001	Supports H5
Contextual support → Perceived transfer gains	0.07	0.183	Not significant

The model explained 31% of the variance in trust calibration, 49% of the variance in collaborative regulation, and 58% of the variance in perceived transfer gains.

### 4.6 Direct, indirect, and total effects

Table 13 reports the direct, indirect, and total effects relevant to the mediation hypotheses. The indirect effect of contextual support on perceived transfer gains through trust calibration was statistically significant, as was the indirect effect of trust calibration on perceived transfer gains through collaborative regulation. Most importantly, the chained indirect pathway from contextual support to perceived transfer gains through trust calibration and collaborative regulation was significant. Because the direct effect of contextual support on perceived transfer gains was not significant, the pattern is more consistent with an indirect process than with a simple direct outcome model.

**Table 13:** Direct, indirect, and total effects.

Effect	Direct	Indirect	Total	95% bootstrap CI for indirect effect
Contextual support → Perceived transfer gains	0.07	0.271	0.341	[0.201, 0.352]
Contextual support → Trust calibration → Perceived transfer gains	NA	0.123	NA	[0.083, 0.176]
Trust calibration → Collaborative regulation → Perceived transfer gains	NA	0.265	NA	[0.196, 0.341]
Contextual support → Trust calibration → Collaborative regulation → Perceived transfer gains	NA	0.148	NA	[0.109, 0.201]
Trust calibration → Perceived transfer gains	0.22	0.265	0.485	[0.196, 0.341]

These results support H6, H7, and H8.

## 5 Discussion

### 5.1 Main finding

The most important finding of the study is that the relationship between supportive educational contexts and students' perceived developmental gains in GenAI collaboration appears to be indirect rather than automatic. Contextual support was associated with perceived gains primarily through multiple indirect pathways involving trust calibration and collaborative regulation rather than through a statistically significant direct path. This result matters because it organizes several strands of GenAI in higher education research into a single process account: contextual guidance shapes judgments, judgments shape regulatory behavior, and regulatory behavior is the most proximal correlate of perceived developmental gain.

### 5.2 Contribution to trust research in education

The study extends educational trust research by arguing that trust calibration is analytically more useful than undifferentiated “trust in AI” when the goal is to explain learning relevant behavior. Prior work has shown that trust influences students' willingness to adopt AI powered educational tools and that trust in educational AI is multidimensional (Nazaretsky et al., 2025; Lelescu et al., 2025). The present model pushes this argument further by locating trust calibration between external context and enacted collaboration. This framing helps clarify why trust calibration should not be treated as a renamed version of critical evaluation or metacognitive monitoring. Its distinctive role is to capture the task level judgment that makes later regulatory action more or less likely. At the same time, the current evidence should not be overstated. The present results are theory consistent structural evidence; they do not prove a temporal mechanism. Stronger evidence for the hinge role of trust calibration would require repeated measurement, behavioral traces of verification, or experimental manipulation of contextual cues.

### 5.3 Contribution to self regulated learning research

The study also contributes to self regulated learning research by showing how collaborative regulation may operate in human AI settings. Existing GenAI studies have suggested that structured or guided AI use can support self regulation, critical thinking, and knowledge construction (Darvishi et al., 2024; Lee et al., 2024; Weng et al., 2024; Zhou et al., 2024). The present model adds theoretical precision by distinguishing between judgment (trust calibration) and enacted process control (collaborative regulation). This matters because learners may understand that AI output requires caution without actually engaging in checking, comparison, or revision. The results therefore support a process account in which regulatory behavior is the nearer predictor of perceived gains than context alone. The directional path from trust calibration to collaborative regulation should therefore be interpreted as one theoretically relevant segment of a broader self-regulatory cycle. At the level of a particular AI output, students' judgments about reliability and task risk can guide whether they verify, compare, or revise the response. At the level of repeated interaction, the consequences of those actions can recalibrate later trust: finding an invented citation may reduce reliance on subsequent factual claims, whereas successfully verifying an explanation may support conditional reliance in a similar task. This recursive interpretation is consistent with cyclical self-regulated learning accounts in which judgment, action, monitoring, and reflection continually provide input to one another (Zimmerman, 2000; Anders and Speltz, 2025; Qi et al., 2025). Thus, the present model does not claim that trust calibration occurs once and is then replaced by collaborative regulation. It isolates the judgment-to-action direction for theoretical parsimony and statistical testing, while recognizing that the complete process is likely to include feedback from regulation to subsequent trust calibration.

### 5.4 Contribution to GenAI in higher education research

Relative to the adoption and AI literacy literatures, the study contributes by clarifying exactly what layer of explanation it addresses. It does not aim to explain whether students adopt GenAI in the first place. It does not aim to offer a complete taxonomy of AI literacy either. Instead, it addresses the missing middle: how supportive environments and task appropriate trust judgments are associated with the quality of student collaboration with GenAI and with students' perceived transfer oriented outcomes. In that sense, the study complements rather than replaces work on adoption, literacy, or ethics. This also explains why the outcome variable is framed conservatively. Perceived transfer gains are meaningful because they represent students' own developmental interpretations of repeated AI collaboration. However, they remain subjective outcomes. The present findings therefore support a structured account of perceived developmental gain, not a claim that objective competence development has already been demonstrated.

### 5.5 Methodological implications

The mixed methods design strengthened the alignment between theory and measurement by using the qualitative phase to generate the constructs before confirmatory testing. This is important in a field where constructs can drift apart quickly and where scale development often lags behind conceptual innovation. Methodologically, the study also improves reporting transparency by making the scale-development logic, descriptive statistics, discriminant-validity checks, and control-variable results explicit. Even so, the methodological limits remain substantial. The quantitative evidence is cross sectional, self reported, and single source. Harman's test is insufficient as a stand alone method bias argument. Although the qualitative phase offers analytic depth and quotation based illustration, the evidence still comes from a bounded sample of Chinese undergraduates and should therefore be interpreted as contextually situated rather than universally generalizable.

### 5.6 Practical implications for trust calibration and collaborative regulation

The findings can be translated into a staged implementation framework for introducing GenAI into university teaching. Before students use an AI tool, instructors can classify course activities by task and output risk and communicate the corresponding expectations for reliance and verification. For example, brainstorming, generating alternative questions, or improving sentence-level expression may be classified as relatively low-risk uses, whereas factual claims, citations, calculations, code, evidence synthesis, and disciplinary interpretation should trigger stronger verification requirements. A course-level AI-use matrix can specify whether GenAI use is permitted, what documentation is required, which sources must be consulted, and which decisions remain the student's responsibility. This makes contextual support concrete by replacing general permission or prohibition with task-specific boundary cues. During instruction, teachers can model trust calibration through comparison activities. Students can be shown contrasting AI responses, such as a fluent response containing a fabricated citation, a cautious response supported by evidence, and a partially correct response requiring revision. They can then classify each output as acceptable for direct use, provisionally useful, requiring independent verification, or unsuitable for use, and justify their decision. A brief calibration worksheet can ask students to record the type of claim, the possible consequence of error, the evidence available, their initial confidence, and the action they selected. Such activities make trust calibration observable as a judgment process rather than presenting trust as a general attitude toward GenAI. Assignments should then convert these judgments into collaborative-regulation routines. Students can be required to submit a concise AI interaction record containing the prompts used, the claims or suggestions considered, the external sources consulted, the parts accepted or rejected, and the revisions made to the final product. For higher-risk claims, instructors can require triangulation against course readings, primary sources, test cases, calculations, or other authoritative evidence. Students may also be asked to compare two prompt versions or two AI outputs and explain why one was more useful. These requirements make prompting, checking, comparison, selective integration, and revision visible without requiring students to disclose every minor interaction. Implementation should be adapted to disciplinary practice. In writing-intensive courses, students can verify citations, distinguish evidence from plausible-sounding assertions, and submit a revision memo explaining how AI-generated text was transformed. In STEM courses, students can test calculations, inspect assumptions, run code against instructor-provided cases, and explain discrepancies between AI output and formal methods. In business or social-science courses, students can evaluate data provenance, compare interpretations, and identify unsupported generalizations. The underlying principle remains the same: the intensity and form of verification should correspond to the risk profile of the task rather than being applied identically to all AI outputs. Assessment should reward the quality of students' decisions and regulatory processes, not merely the quality of the final product or the presence or absence of AI use. Rubrics can include separate criteria for justification of reliance, quality of evidence checking, transparency of revision, and preservation of student ownership. Peer-review activities can further normalize disagreement with confident AI output by asking students to compare verification decisions and identify where different levels of trust were warranted. To examine transfer beyond the immediate assignment, instructors can include a later task involving a different topic, tool, or reduced level of AI support and ask students to identify which evaluation and regulation strategies they reused. Such activities would also help distinguish perceived transfer from objectively demonstrated transfer. Table 14 summarizes how the proposed classroom strategies correspond to the four constructs in the model. These recommendations should be interpreted as theory-informed implementation strategies rather than interventions whose causal effectiveness has been demonstrated by the present cross-sectional data. Future classroom studies should compare these practices with less structured forms of GenAI introduction using behavioral traces, performance measures, and delayed transfer tasks.

**Table 14:** Concrete implementation strategies for GenAI-supported teaching.

Implementation stage	Instructor strategy	Expected student evidence	Primary construct addressed
Course preparation	Develop a task-risk and AI-use matrix specifying permitted uses, verification requirements, documentation, and student responsibility.	Students can identify when AI use is permitted and what level of checking is required for different tasks.	Contextual support
Classroom modeling	Compare accurate, inaccurate, unsupported, and overly fluent AI responses; require students to justify whether each output should be used, verified, revised, or rejected.	A calibration worksheet recording claim type, error risk, available evidence, confidence, and reliance decision.	Trust calibration
Guided practice	Require students to verify selected claims against course readings, primary sources, calculations, test cases, or authoritative external evidence.	A source-check table showing the AI claim, verification source, discrepancy found, and final decision.	Trust calibration and collaborative regulation
Assignment execution	Require a concise record of prompts, alternative outputs, accepted and rejected content, and substantive revisions.	An AI interaction log or revision memo demonstrating iterative prompting, comparison, and selective integration.	Collaborative regulation
Assessment	Include rubric criteria for justification, evidence checking, revision quality, transparency, and preservation of student ownership.	A final product accompanied by process evidence that explains how AI output was evaluated and transformed.	Collaborative regulation
Transfer and reflection	Use a later task with a different topic, tool, or level of AI support and ask students to reuse and explain relevant strategies.	A transfer reflection or delayed performance task demonstrating whether evaluation and regulation strategies were applied in a new context.	Perceived and observed transfer gains

Verification requirements should be adapted to disciplinary conventions and the potential consequences of error. The strategies are theory-informed implications of the present model and have not been tested as a standardized instructional intervention.

### 5.7 Limitations and future research

Several limitations define the interpretive boundary of the present study. The structural model is based on cross sectional self report data, which means that causal or temporal claims should not be drawn. Perceived transfer gains are subjective outcomes rather than objective performance measures. The measurement tool remains an author developed scale and should not be described as a mature, stable instrument without further validation across samples and contexts. The sample was geographically and culturally concentrated: all participants were undergraduates recruited from mainland Chinese universities. This constraint is not solely geographic, because educational norms and institutional arrangements may influence how contextual support, trust calibration, and collaborative regulation are understood and enacted. For example, the effects of teacher guidance and peer modeling may vary across systems with different expectations regarding instructional authority, learner autonomy, academic integrity, and acceptable AI use. Trust judgments and verification practices may also differ according to institutional AI policies, students' prior exposure to GenAI, the dominant language and platform environment, and the availability of alternative information sources. Consequently, the construct meanings may remain broadly relevant across settings while the strength or ordering of the structural relationships differs. Because the present study did not compare cultural or regional groups, its findings should be interpreted as contextually situated evidence rather than as a culture-general model of student–GenAI collaboration.

Future research should therefore proceed in at least four directions. Longitudinal, experimental, and intensive process-based designs are needed to examine the temporal and potentially reciprocal relationship between trust calibration and collaborative regulation. Repeated measures across prompts or task episodes, combined with prompt logs, verification traces, and document revisions, could test whether calibrated trust predicts later regulatory action and whether the outcomes of regulation subsequently update trust. Cross-lagged panel models, dynamic structural equation models, or event-level process analyses would be more appropriate than single-wave SEM for distinguishing temporal precedence, simultaneity, and feedback. Teacher ratings, peer judgments, and objective performance measures should be incorporated to reduce shared method inflation and determine whether the reported developmental gains correspond to externally observable outcomes. Cross-regional and cross-cultural replication is needed to examine whether the construct definitions and structural relationships remain stable across countries, educational systems, disciplines, and task types. Future studies should first establish configural, metric, and scalar measurement invariance before comparing latent means or structural paths across groups. Multi-group structural equation modeling could then test whether relationships involving contextual support, trust calibration, and collaborative regulation vary with institutional AI policy, educational norms, language and platform environments, digital access, or expectations regarding learner autonomy. Future measurement work should examine the predictive validity, test–retest stability, and relationships with external behavioral and performance criteria for the 22-item scale before it is treated as a robust instrument.

## 6 Conclusion

This study proposed and tested a model linking contextual support, trust calibration, collaborative regulation, and perceived transfer gains to explain how university students experience developmental benefits when collaborating with GenAI. Across the mixed methods design, the evidence pointed to a consistent interpretation: supportive educational environments are linked to perceived developmental gains primarily through students' task appropriate trust judgments and their active regulation of human AI interaction. The findings therefore move the discussion beyond simple adoption or exposure arguments and toward a more differentiated explanation of when GenAI supported activity is associated with stronger perceived learning quality. At the same time, the study retains an appropriately cautious interpretive stance by limiting its claims to theory consistent structural evidence and subjective outcomes.

## Data Availability

The original contributions presented in the study are included in the article/supplementary material, further inquiries can be directed to the corresponding author.
